# Rapid and Simultaneous Detection of Aflatoxin B_1_, Zearalenone, and T-2 Toxin in Medicinal and Edible Food Using Gold Immunochromatographic Test Strip

**DOI:** 10.3390/foods12030633

**Published:** 2023-02-02

**Authors:** Jiaying Zhang, Xiujiang Li, Jianhua Xie, Zhibing Huang

**Affiliations:** 1 State Key Laboratory of Food Science and Technology, Nanchang University, No. 235 Nanjing East Road, Nanchang 330047, China; 2 Sino–German Joint Research Institute, Nanchang University, No. 235 Nanjing East Road, Nanchang 330047, China; 3 The First Affiliated Hospital of Nanchang University, Nanchang University, No. 17 Yongwai Main Street, Nanjing West Road, Nanchang 330006, China

**Keywords:** medicinal and edible food, traditional Chinese medicine, mycotoxin, toxin screening, gold immunochromatography

## Abstract

(1) Background: Medicinal and edible food and traditional Chinese medicine have been used to treat various diseases. However, their safety has not been thoroughly assessed. (2) Methods: An immunochromatographic test strip (ICS) was used for the first time to screen some mycotoxins, including aflatoxin B1 (AFB_1_), zearalenone (ZEN), and T-2 toxin, in medicinal and edible food and traditional Chinese medicine. Antibody/nano-gold particle coupling was used with the prepared ICS, and the pH, monoclonal antibody concentration, and antigen amount were optimized. The extraction sample solution was diluted 10 times with phosphate-buffered saline containing 0.5% Tween-20 and 0.05% sodium dodecyl sulfate to remove the complex matrix in medicinal and edible food. (3) Results: Under optimal conditions, the sensitivities of the developed ICS for AFB_1_, ZEN, and T-2 were 0.5, 5.0, and 5.0 ng/mL, respectively. Among the 30 medicinal and edible food samples tested, two samples (both of sand jujube kernels) were positive, and the results were verified by high-performance liquid chromatography and enzyme-linked immunosorbent assay and were consistent with the ICS test results. (4) Conclusions: The ICS could be used for rapid screening and simultaneous detection of mycotoxins at medicinal and edible food storage facilities.

## 1. Introduction

At present, medicinal food and traditional Chinese medicine are widely used as dietary supplements and to treat some diseases; however, the safety of medicinal foods and traditional Chinese medicines is a matter of great concern [1,2]. Crops, plant-derived feed products, foods, and Chinese herbal medicines are rich in polysaccharides, proteins, fats, and other components that are highly susceptible to mycotoxin production [3,4]. Mycotoxins are a class of small molecule secondary metabolites produced by toxin-producing fungi under specific environmental conditions, most of which are produced by filamentous molds, so they are also called mycotoxins. Currently, more than 400 mycotoxins have been reported; the most harmful and common are aflatoxin, vomitoxin, zearalenone, ochratoxin, T-2/HT-2 toxin, fumonisin, penicillin, etc. [5,6,7]. The main toxicity of mycotoxins is carcinogenic, teratogenic, genotoxic, immunotoxic, cytotoxic, etc., which can damage the liver, kidney, nerve tissue, hematopoietic tissue, and skin tissue of humans and animals, thereby causing acute or chronic poisoning of the body [8,9,10]. At present, there is no specific drug for the treatment of mycotoxins, because mycotoxins can contaminate a variety of complex matrices, and can show toxicity at low dose levels, so reliable and sensitive detection methods are required, so it is particularly important to monitor and inspect the contamination of food and traditional Chinese medicines and rescue contaminated food and traditional Chinese medicine.

In order to ensure the safety of medicinal plants, good medical system standards have been established in some countries. The World Health Organization and the European Union set the maximum limit of AFB_1_ to 2.0 µg/kg [11,12] and the United States of America, Japan and the Chinese Pharmacopoeia stipulate that the maximum AFB_1_ content should be 5.0 µg/kg [13,14,15,16]. The Korean Pharmacopoeia limits AFB_1_ content to 10 µg/kg [17]. The toxin content limits for ZEN and T-2 in Chinese medicines have not been set; however, the limit in grains is 60 and 100 µg/kg, respectively. Currently, the most commonly used methods for analysis of mycotoxins are high-performance liquid chromatography (HPLC) and liquid chromatography mass spectrometry (LC-MS). For example, Han et al. [18] established an HPLC method to determine the contents of AFB_1_, AFB_2_, AFG_1_, and AFG_2_ in traditional Chinese medicine. Wang et al. [19] used LC-MS/MS to simultaneously determine 17 mycotoxins in *Pueraria lobata*, and they found that three of 17 samples were simultaneously contaminated by AFB_1_ and T-2 toxin. Although analysis using these methods can detect various mycotoxins sensitively and accurately, this approach cannot be used for onsite detection of mycotoxins in medicinal and edible food and Chinese medicines because of complicated sample preparation procedures, cumbersome detection steps, the need for professional operators, and other inconveniences. The most widely used immunoassays are enzyme-linked immunosorbent assay (ELISA), high performance liquid chromatography (HPLC), and immunochromatography (ICA). However, ELISA kits and HPLC can only detect single toxins in samples one by one, which not only requires professional operators and complex sample preparation steps, but also causes waste of repeated sample processing and consumption of detection time, which greatly increases the difficulty and cost of sample detection [20]. In contrast, colloidal gold immunochromatography test strips have been widely used in the detection of food and drugs due to their advantages of speed, stability, simple and convenient operation, and low cost. Through the improvement of the test strip detection technology, a variety of mycotoxins in the sample can be detected at the same time, which is extremely effective in saving the cost of monitoring and analysis, easy to popularize ICA [21]. Therefore, ICA technology has been widely applied in mycotoxin detection. For example, Hu et al. [22] proposed using an immunochromatographic strip (ICS) to detect AFB_1_ in medicinal plants with a detection limit of 5.0 µg/kg. Di Nardo et al. [23] reported that ICSs can quickly determine corn flour at the same time, and the visual cutoff level is aflatoxin B_1_ 2 μg/kg and fumonisin 1000 μg/kg. Li et al. [24] reported that ICSs could simultaneously detect AFB_1_, ochratoxin A (OTA), and ZEN in grains with detection limits of 0.25, 5.0, and 1.0 ng/mL, respectively. Molinelli et al. [25] reported semiquantitative detection of the T-2 toxin (100 µg/kg) in agricultural products. However, the use of an ICS to detect toxins in medicinal and edible food and traditional Chinese medicines has not been reported.

In food, feed and traditional Chinese medicine mildew often produce a wide variety of mycotoxins, mycotoxin mixed pollution is more prominent, and the harmful effects of different mycotoxins show interaction effect, the definition of interaction effect refers to the two or more mycotoxins at the same time, these mycotoxins on the body show the mutual relationship, including synergistic effect, additive effect, synergistic effect and antagonistic effect research. The establishment of sensitive, accurate, and rapid mycotoxin-mixed pollution detection technology is not only of academic significance. It is also in line with the needs and application prospects of quality and safety testing of food and traditional Chinese medicines and is of great significance to ensuring the safety of humans and animals. Using a single type of mycotoxin assay to test samples in sample immunochromatography test strips requires different types of test strips, more sample volume, and time, which greatly increases the detection cost and manpower and material resources. Therefore, the development of a method that can detect multiple mycotoxins in samples at the same time is extremely effective, saving the cost of monitoring and analysis, and is easy to popularize [26]. 

Therefore, in this study, we aimed to develop an ICS test strip that could quickly and simultaneously screen AFB_1_, ZEN, and T-2 in in medicinal and edible food and Chinese medicines.

## 2. Materials and Methods

### 2.1. Reagents

Anti-AFB_1_, anti-ZEN, and anti-T-2 monoclonal antibodies and antigen pairs were purchased from Wuhan Chundu Biotech (Wuhan, China). AFB_1_, ZEN, and T-2 were obtained from Beijing Bailingwei Co., Ltd. (Beijing, China). FB_1_, deoxynivalenol (DON), OTA, and chloroauric acid (HAuCl_4_) were obtained from Sigma-Aldrich (Milwaukee, WI, USA). Absorbent pad, gold label pad, sample pad, bovine serum albumin (BSA), goat anti-mouse IgG, and conjugate storage buffer were obtained from Jieyi Biotech (Shanghai, China). Nitrocellulose membranes (cat. no. YNHS-120B) were purchased from Guangdong Yineng Membrane Industry Co., Ltd. (Guangdong, China). An enzyme-linked immunosorbent assay (ELISA) kit for T-2 was purchased from Pribolab (Qingdao, China). Other organic and inorganic reagents used were of analytical grade. Thirty samples for medicinal and food purposes were purchased from different regions of China. These include Fallopia multiflora (Nos. 1–4), Codonopsis pilosula (Nos. 5–8), apricot kernel (Nos. 9–10), Zingiber officinale (Nos. 11–12), wild jujube seed (Nos. 13–15), malt (Nos. 16–17), Cassia obtusifolia (Nos. 18–20), the seed of Job’s tears (Nos. 21–27), red lotus seed (No. 28), white lotus seed (Nos. 29–30).

### 2.2. Apparatus

The HGS 501 film was sprayed with a gold labelling machine and prepared using a programmable strip-cutting machine (cat. no. HGS201) from Hangzhou Fenghang Technology Co., Ltd. (Hangzhou, China). A KDC-140HR high-speed refrigerated centrifuge was obtained from Anhui Zhongkejia Scientific Instrument Co., Ltd. (Anhui, China), and aJSM-6701F scanning electron microscope was obtained from Japan JEOL (Tokyo, Japan). An Agilent 1260 liquid chromatography was purchased from Agilent Technologies (Santa Clara, CA, USA). A 759S UV spectrophotometer was obtained from Perkin Elmer (China).

### 2.3. Preparation of Gold Nanoparticles (GNPs)

The Frens method was adopted to prepare spherical GNPs by reducing HAuCl_4_ with trisodium citrate [27]. Briefly, 1.0 mL of 1%(*w*/*v*) HAuCl_4_ aqueous solution was added to 99 mL deionized water. The solution was heated and stirred in a magnetic heating stirrer until small bubbles were generated. Next, a certain amount of 1% (*w*/*v*) trisodium citrate solution was added. The color turned from light yellow to grey and then to a clear wine red, after which heating was performed for an additional 15 min. The prepared colloidal gold was packaged in a glass container and stored in a refrigerator at 4 °C. Ultraviolet-visible spectrometry and scanning electron microscopy (SEM) were used to characterize the prepared GNP solution to determine its particle size, shape, and uniformity.

### 2.4. Optimization of Relevant Parameters of Lateral Flow Assay

It mainly includes the optimization of pH value of gold nano solution, the optimization of monoclonal antibody amount, the optimization of gold standard antibody addition and antigen concentration.

Adjust the pH of the gold nano solution to 5.5, 6.0, 6.5, 7.0, 7.5, and 8.0 with 1% K_2_CO_3_ (*w*/*v*), and then label each beaker with the same concentration of antibody to determine the optimal pH.

Each gold nanoparticle solution was labeled with monoclonal antibodies of different concentrations and volumes, and the optimal amount of monoclonal antibody was judged by the color development of C and T lines on the test strip.

The optimal pH value and optimal monoclonal antibody amount determined in the above experiment were labeled, and 4 groups of different gold standard antibody additions and 4 groups of different antigen concentrations were selected for orthogonal experiments to determine the optimal gold standard antibody amount and optimal antigen concentration. In addition, the loading solution was optimized.

### 2.5. Assembling of Lateral Flow Assay

**Figure 1:**
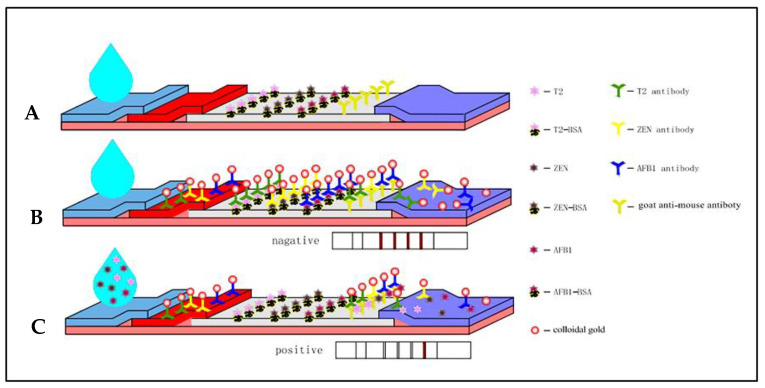
Schematic of the immunochromatographic strip (ICS) for the simultaneous detection of AFB_1_, ZEN, and T-2 (**A**). Negative test (**B**). Positive test (**C**). Here, BSA is bovine serum albumin.

As shown in Figure 1, the nitrocellulose film, absorbent pad, gold label pad, and sample pad were glued to the bottom of a polyvinyl chloride plate. Next, rabbit antimouse IgG antibody, AFB_1_-BSA, ZEN-BSA, and T-2-BSA were sprayed on the nitrocellulose membrane as the control line (C), test line 1 (T1), test line 2 (T-2), and test line 3 (T-3). Membranes were then assembled into test strips and placed in a drying oven at 35 °C for 2 h. The HGS201 programmable strip cutting machine was used to cut dried test strips into the size required for the experiment (60 × 4 mm). Strips were placed in a plastic Ziplock bag and stored at a constant temperature of 25 °C until use. 

### 2.6. Principles of the GNP-Based ICS Assay

The test strip is based on the principle of competition. As shown in Figure 1, when the sample solution containing target AFB_1_, ZEN, and T-2 drops on the sample pad and migrates to the conjugate pad through capillary action, GNP conjugates (GNP-anti-AFB_1_, GNP-anti-ZEN, GNP-anti-T-2) are formed on the gold-labeled antibody complex fixed on the conjugate pad. When the solution passed through the conjugate pad, the antigen on the T-line could not retain the GNP-mAb occupying the binding site. As a result, the antigen concentration was inversely proportional to the color of the band on the T-line. As the sample solution continued to migrate, the free GNP conjugate and the complex of antigen and GNPs were captured by the secondary antibody on the C line. However, if there was no target toxin in the sample solution, the antigen on the T-line would capture the GNP conjugate, and the T-line developed color. The excess GNP conjugate would then be captured by the secondary antibody.

Thus, the presence of color at the C-line could be an indicator of whether the ICS was functional, and the appearance of color at the T-line could be used to determine the minimum visual detection limit of the target toxin and whether there was target toxin in the sample being tested.

### 2.7. Preparation of Monoclonal Antibodies (mAbs) Labelled with GNPs

The preparation of GNP-mAbs has been reported previously [28,29,30]. Briefly, the pH was adjusted with 1% K_2_CO_3_, and mAbs at different concentrations were added according to the difference in the colloidal gold solution for labelling. The GNP-mAbs were then added to the test strips, and color development was monitored the C\T line of the antibody concentration.

Under optimal pH conditions, anti-AFB_1_, anti-ZEN, or anti-T-2 mAbs were added for nonspecific binding of GNPs to the toxin antibody site. After stirring for 30 min, 10% BSA (100 µL) was added, and stirring was continued for 15 min to block nonspecific binding on the surface of the GNPs. Finally, the mixed solution was centrifuged at 4 °C and 8000× *g* for 20 min. After discarding the clear supernatant, the GNP-labelled pellet was resuspended in conjugate storage buffer and stored in a refrigerator at 4 °C.

### 2.8. Determination of the Specificity of the Test Strips

Test strips are prepared for the determination of toxins in buffer solutions. Different mycotoxins were selected dropwise added to the immunochromatography test strip to observe the results, the color of the T line in the test area of the test strip was used to determine whether the test strip showed cross-reactivity with other toxins, if the T line did not develop color, the prepared test strip had good specificity, and if the T line developed color, the prepared strip had poor specificity.

### 2.9. Determination of the Visual Detection Limit of the Test Strips

We prepared a series of solutions containing different concentration gradients of toxins AFB_1_, ZEN, and T-2 and dropped the solution on the test strip. We then determined whether the T line in the test area of the test strip disappeared using visual inspection to determine the visual inspection limit. The lowest concentration of toxin buffer at which the observable T line disappeared was the visual detection limit.

### 2.10. Real Sample Detection

Medicinal and edible food and traditional Chinese medicines were collected from different regions of China. The samples were crushed with a high-speed grinder and sieved through a 100-mesh sieve. The quarter method was used to obtain 5.0 g, and 5.0 mL methanol was added for extraction. The samples were then mixed with a vortex mixer for 5 min. After centrifugation, the precipitate was discarded, and the clear supernatant was collected and filtered twice with a 0.22-μm filter membrane. Finally, samples were diluted with phosphate-buffered saline (PBS; pH7.4) containing 0.5% Tween-20 (PBST) and 0.05% sodium dodecyl sulfate. In the ICS test sample dilution, we observed after 10 min whether the sample contained the detected toxin and whether the concentration exceeded the detection limit.

In order to verify the accuracy of the test method, the contents of AFB_1_ and ZEN in the sample were determined by HPLC, and the content of T-2 was determined by ELISA. The test methods were performed according to the Chinese National Standard.

## 3. Results and Discussion

### 3.1. Preparation of GNPs

GNPs with different particle sizes can alter the stability and sensitivity of ICSs, and the particle size of GNPs is related to the addition of a reducing agent (trisodium citrate). When the prepared GNPs are larger, the formed GNP-mAb conjugate is more unstable; conversely, when the particle size is smaller, the sensitivity is lower. According to the literature, when the particle size of spherical GNPs is approximately 25 nm, GNPs are considered suitable for conjugation to proteins. The color of the colloidal gold solution prepared in our group was bright red, clear, and transparent and showed good uniformity. We chose the GNP solution containing 1.4 mL trisodium citrate, and in the ultraviolet-visible spectrum, the maximum absorption was observed at 520 nm [29,31].

### 3.2. Optimization of Parameters

**Figure 2:**
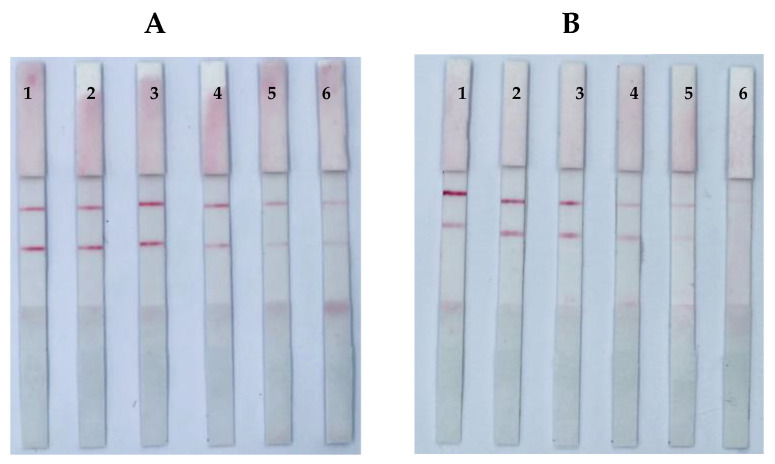
Optimization of pH. ICSs of ZEN (**A**), and T-2 (**B**). The pH values (labels 1–6) were 5.5, 6.0, 6.5, 7.0, 7.5, and 8.0, respectively.

The key parameters for the successful preparation of GNP-mAb probes were pH and the mAb concentration in the GNP solution; these factors determined the stability of the GNP-mAb solution and the sensitivity of the ICS. In this study, 1% K_2_CO_3_ was used to adjust the pH value, and accurate pH test paper was used to determine the optimal pH value at which the mAbs easily coupled to the GNP surface. Notably, we found that within the pH range of 5.5–8.0, the optimal pH values for AFB_1_, ZEN, and T-2 were 6.0, 6.5, and 6.0, respectively (Figure 2).

**Figure 3:**
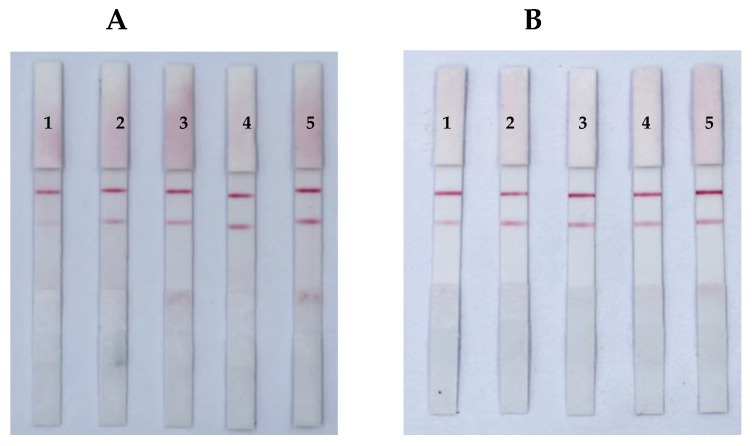
Optimization of antibody concentrations. ICSs of ZEN (**A**), and T-2 (**B**). Amount of anti-body (labels 1–5) were 1.9, 2.1, 2.3, 2.5, and 2.7 μg, respectively.

Next, we evaluated the optimal mAb concentration by adding different amounts of GNP-mAb at the optimal pH value. As the amount of mAb increased (1.9–3.1 μg), the color of the ICS band gradually changed. Thus, we chose 2.7 [31], 2.5, and 2.1 μg for anti-AFB_1_, anti-ZEN, and anti-T-2 mAbs, respectively; at these concentrations, the C/T color intensity of the ICS was most consistent (Figure 3).

**Figure 4:**
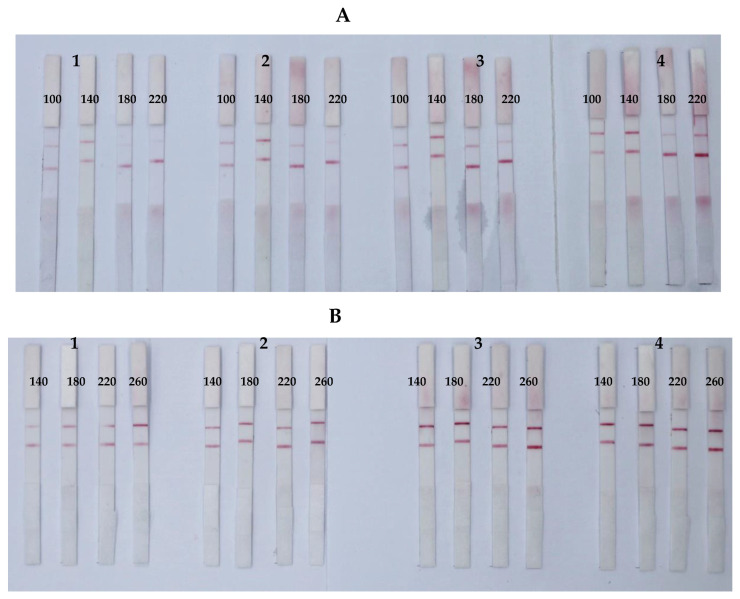
Optimization of the amount of GNP-labeled antibody and the concentration of antigen ICSs of ZEN (**A**), and T-2 (**B**). The amounts of GNP-labelled antibodies were 1, 2, 3, and 4 μL. The concentrations of ZEN-BSA were 100, 140, 180, and 220 μg/mL, and the concentrations of T-2-BSA were 140, 180, 220, and 260 μg/mL.

By adding a certain amount of GNP-mAb solution, an orthogonal method was used to react the GNP-mAbs with different concentrations of antigens in order to select the lowest amount relative to the capture antigen and mAb to optimize the sensitivity of the ICS. The results (Figure 4) showed that the optimal GNP-mAb solution volumes were 3, 2, and 1 μL, corresponding to the optimal antigen concentrations of 180, 140, and 180 μg/mL for AFB_1_ [31], ZEN, and T-2, respectively.

### 3.3. Optimization of Loading Solution of the ICS

**Figure 5:**
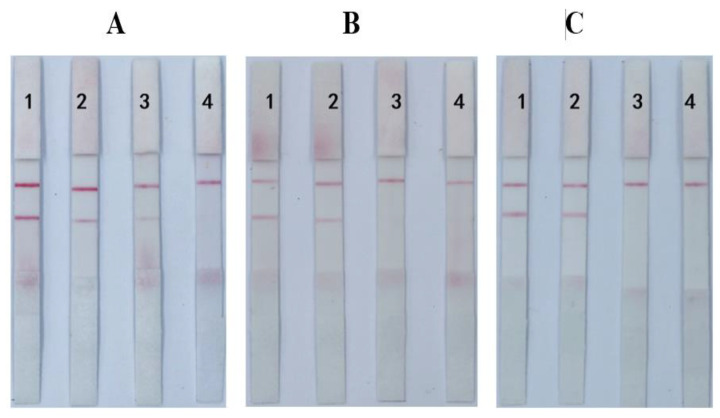
Optimization of loading solution of the ICS. (**A**) AFB_1_ test strips; (**B**) ZEN test strips; (**C**) T-2 test strips. Note: 1–4—separate loading solutions without 0.005% SDS; loading solution containing 0.005% SDS; standard solution without 0.005% SDS; standard solution containing 0.005% SDS.

As shown in Figure 5A1, when 100 μL of loading solution (20% PBST) and 100 μL of 0.5 ng/mL standard (Figure 5A3) are added to the prepared monotoxin AFB_1_ spherical nanogold test strip and 100 μL of 0.5 ng/mL standard (Figure 5A3), both C and T lines are colored, which may be due to the nonspecific adsorption caused by the electrostatic interaction between the gold standard antibody on the strip and the capture antigen during the detection process, and 0.005% anionic surfactant is added to the sample loading solution [32]. As shown in Figure 5A2, the addition of 100 μL of sample loading solution containing 0.005% SDS (20% PBST) was T line chromogenic and significantly blocked when 100 μL of 0.5 ng/mL standard containing 0.005% SDS was added, and the results showed that the addition of 0.005% SDS effectively eliminated nonspecific adsorption between the gold standard antibody and the capture antigen. The addition of 100 μL of 0.005% SDS loading solution (Figure 5B1,B2) and 0.005% SDS loading solution (Figure 5B2,C2), respectively, on single-toxin ZEN and T-2 strips showed the effect of the addition of 0.005% SDS loading solution on the detection of ZEN and T-2, respectively. Figure 5B3,B4,C3,C4 show that the addition of a loading solution containing 0.005% SDS to the standard has no effect on blocking. Therefore, the loading solution containing 0.005% SDS can be used when testing AFB_1_, ZEN, and T-2 simultaneously.

### 3.4. Sensitivity, Specificity, and Stability of the ICS

**Figure 6:**
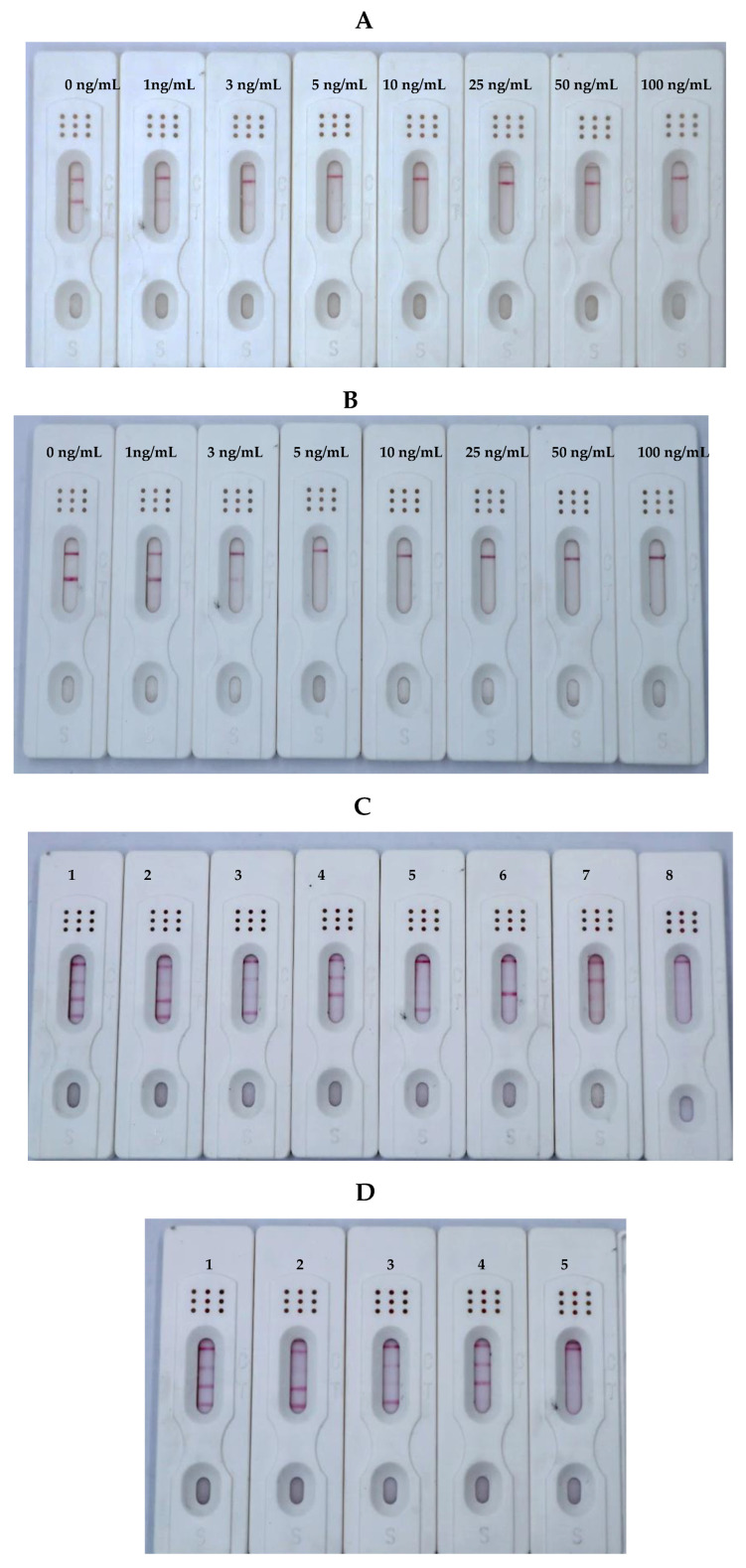
Sensitivity of the immunochromatographic strip (ICS) to ZEN (**A**), and T-2 (**B**). (**C**) Simultaneous detection of sensitivity for AFB_1_, ZEN, and T-2. Blank control (1); 0.5 ng/mL AFB_1_ (2); 5.0 ng/mL ZEN (3); 5.0 ng/mL T-2 (4); 0.5 ng/mL AFB_1_ + 5.0 ng/mL ZEN (5); 0.5 ng/mL AFB_1_ + 5.0 ng/mL T-2 (6); 5.0 ng/mL ZEN + 5.0 ng/mL T-2 (7); 0.5 ng/mL AFB_1_ + 5.0 ng/mL ZEN + 5.0 ng/mL T-2 (8). (**D**) Storage of the ICS for 120 days. Blank control (1); 0.5 ng/mL AFB_1_ (2); 5.0 ng/mL ZEN (3); 5.0 ng/mL T-2 (4); 0.5 ng/mL AFB_1_ + 5.0 ng/mL ZEN + 5.0 ng/mL T-2 (5).

Sensitivity is the response degree of the test strip to changes in the content to be tested. By adding different concentrations of toxin buffer to observe the disappearance of the T line. Thus, we prepared a series of standard solutions with different gradients to determine the sensitivity of the ICS. For a single toxin, when the AFB_1_ standard solution was applied to the ICS test paper, the T line in the AFB_1_ test area gradually disappeared, and complete absence of the T line was observed at 0.5 ng/mL [31]. When the ZEN standard solution (Figure 6A) or T-2 standard solution (Figure 6B) was applied to the ICS test strip, the T line completely disappeared at 5.0 ng/mL. For the toxin mixture, as shown Figure 5C, the sensitivities of the ICSs for AFB_1_, ZEN, and T-2 were 0.5, 5.0, and 5.0 ng/mL, respectively.

**Figure 7:**
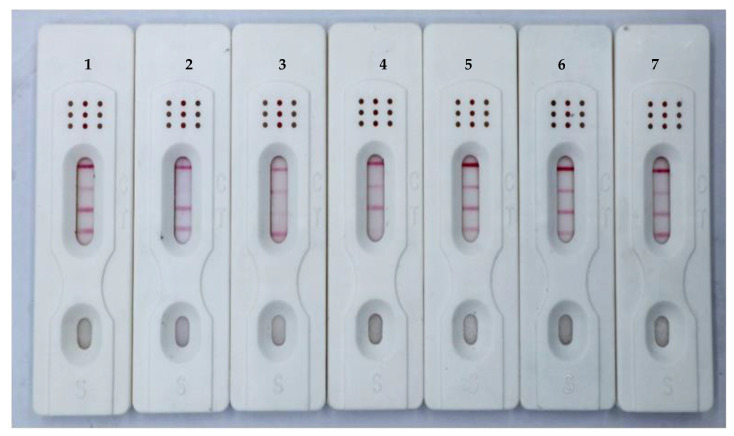
Specificity of ICS (Toxin standard species and concentrations). The concentrations of the toxins were as follows: blank control (1), AFB_1_, 0.5 ng/mL (2); ZEN, 5.0 ng/mL (3); T-2, 5.0 ng/mL (4); FB, 100 ng/mL (5); DON, 100 ng/mL (6); OTA, 100 ng/mL (7).

As shown in Figure 7, six common toxins (AFB_1_, ZEN, T-2, FB1, DON, and OTA) were detected by the ICSs in this study. The ICS did not cross-react with the other three toxins, indicating that the ICS was highly specific for AFB_1_, ZEN, and T-2.

The assembled ICS was packaged in a plastic Ziplock bag and placed at 25 °C for 120 days to detect color changes at the C and T lines of the blank control group and the positive test group. The results showed that the ICS blank control group showed a similar color to the unsaved blank group after storage for different times. Moreover, the positive test group and the blank group showed the same color (Figure 6D). Thus, our findings demonstrated that the storage method used in this study had only minor effects on the color development and sensitivity of the ICS and that ICS had good stability.

### 3.5. Analysis of Mycotoxins in Real Samples

**Figure 8:**
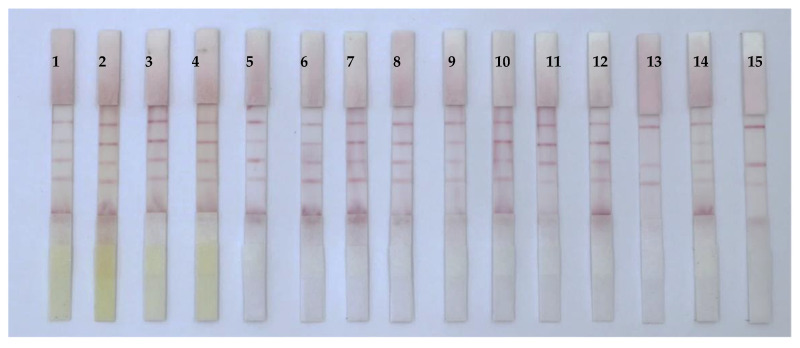

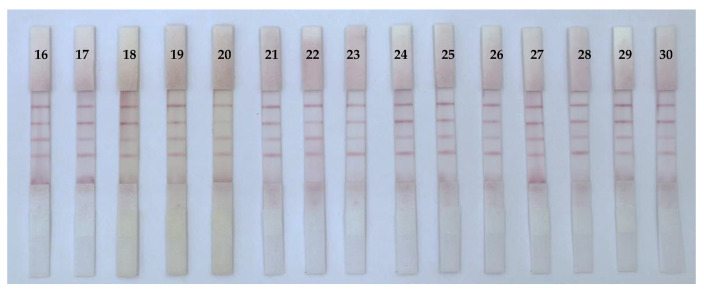
Simultaneous detection of AFB_1_, ZEN, and T-2 in various samples using immunochromatographic strip (ICS).

Thirty different medicinal and edible food and traditional Chinese medicine samples were purchased from local pharmacies and online sales. After extraction with methanol, the extract was diluted 10 times with PBST, and the test sample solution was evaluated using the developed ICS. The results (Figure 8) showed that sample nos. 13 and 15 (both of sand jujube kernels) were positive. At the same time, the samples were tested using HPLC and ELISA (Table 1 and Table 2). When mycotoxins AFB_1_, ZEN, and T-2 were present in the samples at a concentration lower than 5.0 μg/kg, the T line of 50 μg/kg test area showed a red band, indicating negative; otherwise, the results were positive. These findings demonstrated that the ICS results were consistent with the findings from HPLC and ELISA detection. Overall, the developed ICS could simultaneously detect AFB_1_, ZEN, and T-2 and may have applications in the detection and analysis of medicinal and edible food and traditional Chinese medicine samples.

## 4. Conclusions

In this study, we developed an ICS based on the principle of small molecule competition. The developed ICS can simultaneously detect mycotoxins AFB_1_, ZEN, and T-2 present in food and traditional Chinese medicine. We optimized pH, antigen concentration, and antibody concentration, and ICS can detect in as little as 10 min. In addition, ICS has good specificity and stability, and the sensitivity of AFB_1_, ZEN, and T-2 is 0.5, 5.0, and 5.0 ng/mL, respectively, which meets the restriction standards of Chinese pharmacopoeia for traditional Chinese medicines. Negative compliance rate 100%, positive compliance rate 95%. Importantly, we found that the ICS test results were consistent with the results of HPLC and ELISA, indicating that the ICS developed in this study can be used for simultaneous onsite detection of AFB_1_, ZEN, and T-2 toxins in food and traditional Chinese medicine, which is in line with the needs and application prospects of agricultural product quality and safety testing, and is of great significance for ensuring the safe consumption of food and traditional Chinese medicine. 

In this paper, only 25 nm spherical gold nanoparticles were used to prepare the test strips, and the optimization of the sensitivity of immunochromatographic test strips by gold nanoparticles of other particle sizes was not studied. This condition can then be optimized to further improve the sensitivity of the immunochromatography strip. The test strip prepared in this paper can only be qualitatively detected and cannot be quantitatively analyzed, and it is hoped that the immunochromatography test strip can detect the above three mycotoxins semi-quantitatively or quantitatively at the same time.

## Figures and Tables

**Table 1:** ICS and ELISA detection results of ZEN in traditional Chinese medicine samples.

Chinese Medicine	ICS	HPLC	Chinese Medicine	ICS	HPLC
	(*n* = 3)	(μg/kg)		(*n* = 3)	(μg/kg)
1	-,-,-,	ND	16	-,-,-,	ND
2	-,-,-,	ND	17	-,-,-,	ND
3	-,-,-,	ND	18	-,-,-,	ND
4	-,-,-,	ND	19	-,-,-,	ND
5	-,-,-,	ND	20	-,-,-,	ND
6	-,-,-,	ND	21	-,-,-,	ND
7	-,-,-,	ND	22	-,-,-,	ND
8	-,-,-,	ND	23	-,-,-,	ND
9	-,-,-,	ND	24	-,-,-,	ND
10	-,-,-,	ND	25	-,-,-,	ND
11	-,-,-,	ND	26	-,-,-,	ND
12	-,-,-,	ND	27	-,-,-,	ND
13	-,-,-,	ND	28	-,-,-,	ND
14	-,-,-,	ND	29	-,-,-,	ND
15	-,-,-,	ND	30	-,-,-,	ND

ND, Not detected; -, Negative.

**Table 2:** ICS and ELISA detection results of T-2 in traditional Chinese medicine samples.

Chinese Medicine	ICS	ELISA	Chinese Medicine	ICS	ELISA
	(*n* = 3)	(μg/kg)		(*n* = 3)	(μg/kg)
1	-,-,-,	33.49	16	-,-,-,	40.09
2	-,-,-,	ND	17	-,-,-,	31.28
3	-,-,-,	ND	18	-,-,-,	36.07
4	-,-,-,	17.16	19	-,-,-,	41.53
5	-,-,-,	29.26	20	-,-,-,	38.14
6	-,-,-,	29.41	21	-,-,-,	ND
7	-,-,-,	40.97	22	-,-,-,	26.27
8	-,-,-,	12.13	23	-,-,-,	4.41
9	-,-,-,	10.12	24	-,-,-,	6.93
10	-,-,-,	19.55	25	-,-,-,	3.40
11	-,-,-,	16.09	26	-,-,-,	2.75
12	-,-,-,	12.07	27	-,-,-,	3.87
13	-,-,-,	10.88	28	-,-,-,	36.76
14	-,-,-,	13.71	29	-,-,-,	34.31
15	-,-,-,	28.15	30	-,-,-,	25.76

ND, Not detected; -, Negative.

## Data Availability

The data that support the findings of this study are available from the corresponding author upon reasonable request.
